# Preliminary Estimation of Deoxynivalenol Excretion through a 24 h Pilot Study

**DOI:** 10.3390/toxins7030705

**Published:** 2015-02-25

**Authors:** Yelko Rodríguez-Carrasco, Jordi Mañes, Houda Berrada, Guillermina Font

**Affiliations:** Area of Toxicology, Department of Public Health, Faculty of Pharmacy, University of Valencia, Burjassot 46100, Spain; E-Mails: yelko.rodriguez@uv.es (Y.R.-C.); jordi.manes@uv.es (J.M.); guillermina.font@uv.es (G.F.)

**Keywords:** deoxynivalenol, duplicate diet study, GC-MS/MS, urine, risk characterization

## Abstract

A duplicate diet study was designed to explore the occurrence of 15 *Fusarium* mycotoxins in the 24 h-diet consumed by one volunteer as well as the levels of mycotoxins in his 24 h-collected urine. The employed methodology involved solvent extraction at high ionic strength followed by dispersive solid phase extraction and gas chromatography determination coupled to mass spectrometry in tandem. Satisfactory results in method performance were achieved. The method’s accuracy was in a range of 68%–108%, with intra-day relative standard deviation and inter-day relative standard deviation lower than 12% and 15%, respectively. The limits of quantitation ranged from 0.1 to 8 µg/Kg. The matrix effect was evaluated and matrix-matched calibrations were used for quantitation. Only deoxynivalenol (DON) was quantified in both food and urine samples. A total DON daily intake amounted to 49.2 ± 5.6 µg whereas DON daily excretion of 35.2 ± 4.3 µg was determined. DON daily intake represented 68.3% of the established DON provisional maximum tolerable daily intake (PMTDI). Valuable preliminary information was obtained as regards DON excretion and needs to be confirmed in large-scale monitoring studies.

## 1. Introduction

Cereals are the most important source of food for both direct human consumption and livestock production. In fact, the latest published data by the Food and Agricultural Organization (FAO) reported an annually cereal global consumption (excluding beer) of 146.7 kg/capita [1]. Mycotoxins are secondary fungi metabolites produced in several commodities that could exert toxic effects on animals and humans [2] and mycotoxin contamination of cereals is also frequently reported as a public health threat [3,4]. Acute exposures to mycotoxins are related to gastrointestinal manifestations such as diarrhea, vomiting and melena, while chronic exposures and the most worrisome one are related to degenerative effects on the immune, neural and reproductive systems. Chronic exposure to some mycotoxins are also related to cancer induction [5]. Mycotoxins have also been classified as priority food contaminants by the Global Environment Monitoring System-Food Contamination Monitoring Assessment Programme (GEMS/Food) of the World Health Organization (WHO) [6]. Moreover, mycotoxins were the main hazards in the European Union with 425 border rejection notifications as highlighted its annual report for 2012, according to the Rapid Alert System for Food and Feed [7].

Among the wide number of mycotoxins, zearalonene and trichothecenes (type A mainly represented by T-2 and HT-2 toxins, and type B by deoxynivalenol (DON)) constitute one of the largest groups of mycotoxins produced by *Fusarium* in cereal grains [8,9]. Mycotoxin production in agricultural crops can occur at various stages in the food chain like pre-harvest, harvest, drying and storage. In addition, mycotoxins tend to persist during the transformation and processing of contaminated plants and are also still reported in cooked and sterilized food [10].

The European Commission (EC) has set maximum limits for some mycotoxins in foodstuffs [11,12] and the Joint FAO/WHO Expert Committee on Food Additives (JEFCA) established maximum tolerable daily intakes to ensure food safety [13]. According to the guidelines published by the WHO, the basic approaches that can be used to determine the intake of a food contaminant include: total diet studies, duplicate diet studies, and selective studies of individual foods, which combine food consumption patterns and contamination level [14]. Duplicate diet studies may be a good alternative to total diet studies, especially when there are important economical limitations to perform a suitable total diet studies. In addition, duplicate diet studies are particularly interesting to consider not only basic cooking methods, but real cooking, something essential when evaluating the dietary intake in specific individuals, countries or regions [15]. A step forward in the individual exposure assessment could be provided by biomarkers measured in biological fluids. The identification of mycotoxins and their main metabolized products in urine could therefore serve as such biomarkers and could facilitate effective exposure assessment [16,17].

Knowledge of mycotoxins *in vivo* metabolism in humans has been rarely investigated and it is fundamental to carry out some studies which serve as an approach to assess the exposure. In this line, some experiments were designed to provide tentative information about the human *in vivo* metabolism of major *Fusarium* mycotoxins. For instance, Mirocha *et al*. [18] studied the zearalenone metabolite pattern in 24h urine after ingestion of 100 mg zearalenone at once by one volunteer. Similarly, Warth *et al*. [19] carried out a study to investigate the human deoxynivalenol and zearalenone *in vivo* metabolism through the analysis of urine samples obtained from one volunteer following a naturally contaminated diet containing 138 µg DON and 10 µg ZON over a period of four days. Thus, this preliminary work was designed as the previously ones to provide basic information about the human *in vivo* metabolism and serves for the purpose to develop a method which might principally suited as a screening tool. The dietary intake of 15 mycotoxins was studied in a single individual using the duplicate diet approach. All food products as prepared, served and consumed were analyzed. The occurrence of mycotoxins and metabolites was also evaluated in the 24-hour urine collection and expressed by µg/g of creatinine. The aims of the study were to investigate the daily mycotoxin intake through complete cooked meals, to estimate the mycotoxin urinary excretion, and to carry out a risk characterization approach for the participant.

## 2. Results and Discussion

### 2.1. Method Performance

Regression equations were obtained using eight standard concentrations on the abscissa and the area of the chromatogram peaks as vertical coordinates. Linear range was tested at eight concentration levels in triplicate from LOQ to 100 µg/kg. Relative standard deviations among the triplicate were below 5% at all calibration curve points. The determination coefficients (*R*^2^) of all analytes were >0.995. The matrix effect was observed (from 83% to 91%), and thus matrix-matched calibration curves were used for quantification purposes. Apparent recovery for each mycotoxin was determined in composite, beer and urine samples spiked at low and high level (Table 1).

Recoveries values obtained, from 68 to 108%, were in agreement with the range set in legislation [20]. Corrections based on recovery percentages were not performed. A precision study was performed by determining the repeatability (intra-day precision) (*n =* 6) and reproducibility (inter-day precision) (*n =* 4), and was fulfilled in conformity with the described criteria in current legislation. Intra-day and inter-day precision were lower than 12 and 15%, respectively in the assayed matrices. The sensitivity of the method was expressed in terms of limits of detection (LOD) and limits of quantitation (LOQ). LOD and LOQ values were calculated from spiked samples chromatograms based on a signal to noise ratio of 3:1 and 10:1, respectively (Table 1). LODs varied in the following ranges of 0.6–5 µg/kg, 0.05–8 µg/kg and 0.1–4 µg/kg for composite, beer and urine respectively. LOQs varied in the following ranges of 1.2–10 µg/kg, 0.1–16 µg/kg and 0.2–8 µg/kg, for composite, beer and urine respectively, which guaranteed quantitation at low ppb-level.

**Table 1:** Method performance for fifteen mycotoxins and metabolites in composite diet, beer and urine.

Analyte	Composite diet				Beer				Urine			
	REC± RSD (%)		LOD (µg/kg)	LOQ (µg/kg)	REC ± RSD (%)		LOD (µg/kg)	LOQ (µg/kg)	REC ± RSD (%)		LOD (µg/kg)	LOQ (µg/kg)
	Low level ^a^	High level ^c^			Low level ^b^	High level ^c^			Low level ^a^	High level ^c^		
DOM-1	87 ± 7	93 ± 5	0.6	1.2	73 ± 6	77 ± 8	0.1	0.2	84 ± 2	86 ± 4	0.2	0.5
DON	89 ± 5	91 ± 6	0.6	1.2	75 ± 9	83 ± 9	0.05	0.1	96 ± 4	94 ± 8	0.1	0.2
3-ADON	95 ± 4	90 ± 7	0.6	1.2	82 ± 6	80 ± 5	2	4	92 ± 5	94 ± 5	0.2	0.5
FUS-X	84 ± 5	89 ± 3	2.5	5	98 ± 8	93 ± 9	8	16	95 ± 3	90 ± 6	2	4
DAS	103 ± 3	99 ± 6	2.5	5	78 ± 6	82 ± 5	4	8	89 ± 4	84 ± 8	1	2
NIV	79 ± 6	82 ± 5	1.2	2.5	77 ± 12	81 ± 9	0.5	1	87 ± 3	93 ± 6	0.5	1
NEO	97 ± 8	92 ± 5	2.5	5	83 ± 8	88 ± 6	2	4	93 ± 5	94 ± 5	0.2	0.5
HT-2	93 ± 7	89 ± 8	1.2	2.5	97 ± 9	93 ± 4	2	4	96 ± 4	91 ± 8	1	2
T-2	84 ± 9	90 ± 6	2.5	5	108 ± 7	97 ± 8	4	8	102 ± 6	94 ± 9	0.5	1
ZAN	85 ± 5	90 ± 4	5	10	68 ± 9	73 ± 9	8	16	72 ± 7	74 ± 8	4	8
α-ZAL	72 ± 8	79 ± 8	5	10	70 ± 6	78 ± 7	4	8	79 ± 5	82 ± 5	4	8
β-ZAL	79 ± 6	77 ± 6	5	10	73 ± 8	79 ± 8	4	8	77 ± 8	74 ± 6	4	8
ZON	87 ± 8	84 ± 7	2.5	5	71 ± 5	78 ± 6	8	16	81 ± 5	84 ± 7	3	6
α-ZOL	83 ± 9	80 ± 7	2.5	5	78 ± 6	83 ± 4	2	4	88 ± 2	93 ± 5	1	2
β-ZOL	77 ± 6	78± 9	2.5	5	74 ± 8	73 ± 8	4	8	80 ± 6	84 ± 9	2	4

^a^ Spiking level: 10 µg/kg; ^b^ Spiking level: 20 µg/kg; ^c^ Spiking level: 100 µg/kg.

### 2.2. Deoxynivalenol Reduction during Cooking

**Figure 1:**
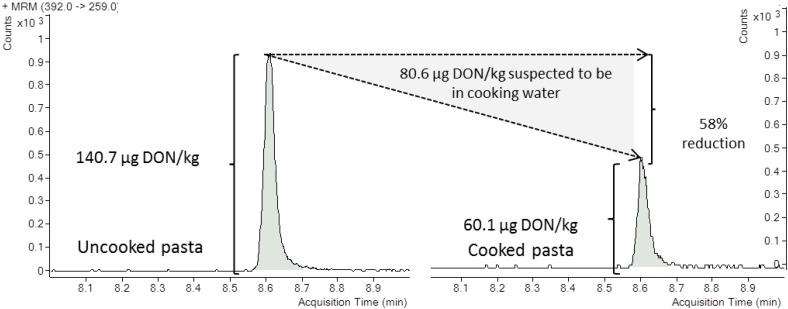
Selected reaction monitoring (SRM) chromatograms of the naturally deoxynivalenol (DON) contaminated pasta before and after culinary treatment at 140.7 µg/kg and 60.1 µg/kg, respectively, on a dry weight basis, and percentage of mycotoxin reduction.

Mycotoxin analyses of pasta (spaghetti) and whole-wheat pasta (little stars) were carried out prior the cooking step and then cooked after the drying process. Analyses were performed in triplicate. The purpose was to evaluate the percentage of mycotoxin reduction during food preparation procedure. Most of the exposure assessment approach to contaminants has been carried out based on un-cooked food, and thus assuming some uncertainty in the reported data. In this work, not only cooked meals but also the regular cooking practices and the serving size were taking into account. The aim was not to serve as a representative data of percentage of reduction for wheat-based products, but to minimize the uncertainty of the obtained data in order to allow a closer exposure assessment approach. A reduction of 13% and 58% was obtained for whole-wheat pasta and pasta, respectively after culinary treatment (Figure 1). That difference could be attributed to the distinct serving. While the cooking water is removed in spaghetti serving, little stars are consumed as a soup and thus, the amount of mycotoxin intake in little stars was higher than in spaghetti. The percentage of reduction obtained in this work were comparable than those reported Visconti *et al*. [10] in a larger study showing average DON reduction levels of 40%. Moreover, they indicated that the amount of DON retained by cooked pasta consistently decreased by increasing the pasta/water ratio during cooking.

### 2.3. Deoxynivalenol Content in Food

All analyzed meals were wheat-based. Within the 15 mycotoxins targeted in the proposed procedure only DON was detected in the analyzed food commodities. This high DON prevalence was highlighted before in cereal and their derivative products by several authors [5] as well as in urine samples [21]. Quantified DON (*n =* 3) in the different food items are presented in Table 2. The food items presenting the highest concentrations of DON were whole-wheat pasta and toasts with average values of 272.4 and 190.6 µg/kg, respectively. Despite beer was the matrix with the lowest DON content, it was the food item with the highest significant contribution to DON intake (28.6 µg) because of the consumption data (0.83L). A total DON daily intake throughout the consumption of the followed diet was estimated in 49.2 ± 5.6 µg. Mean DON contents of the studied food matrices were in line with results reported by other authors [22,23,24,25].

**Table 2:** Overview of contamination level, consumption data and mycotoxin intake contribution of the food items consumed.

Time of consumption	Food	Consumption (g/day)	Mean DON ± SD (µg/kg)	Mean DON intake (µg)
8 am	Toast	45	190.6 ± 2.3	8.7
11 am	Breadsticks	30	49.7 ± 4.6	1.5
2 pm	Pasta	67 ^a^	58.2 ± 2.7	3.9
7 pm	Wheat beer	500	36.4 ± 1.8	18.1
8 pm	Beer	330	32.1 ± 6.2	10.5
10 pm	Whole-wheat pasta	24 ^a^	272.4 ± 5.9	6.5
				Σ DON Intake: 49.2 µg
	Composite ^b^	166	120 ± 7.3	19.9 µg

^a^ on a dry weight basis; ^b^ no beverages included.

The estimated levels of mycotoxins in the composite are also presented in Table 2. DON was quantified at mean level of 120.5 ± 7.3 µg/Kg. A mean DON intake of 19.9 µg was obtained throughout the solid food items consumed. The composite’s result was in line with the 20.5 µg of DON obtained from the sum of each food item. Thus, composite could be presented as an alternative tool to reduce sampling and analysis cost. However, the resulting information concerning contaminants in each selected food item will be missed.

### 2.4. DON Content in Urine

Quantifiable amounts of mycotoxins, and its toxin derivatives that result from its biotransformation, are expected to be found in urine. For instance, DON can be metabolized within the intestinal lumen by gut microbiota, generating the less toxic de-epoxy metabolite known as DOM-1. Further metabolism of DON and ZON to a less toxin metabolite involves the addition of glucuronic acid, catalyzed by UDP-glucuronyltransferase [26,27]. As regards mycotoxin conjugation, the uncertainness exists, since it has been related individual difference in the enzymatic system. On the other hand, Turner *et al*. [28,29] suggested that un-conjugated DON can also persist and it can be excreted in urine. In this line, this work was focused on the investigation of un-metabolized DON as a preliminar step since it needs to be extended in the future to understand the relation between the mycotoxin intake and mycotoxin levels in urine, both metabolized and un-metabolized fractions.

**Figure 2:**
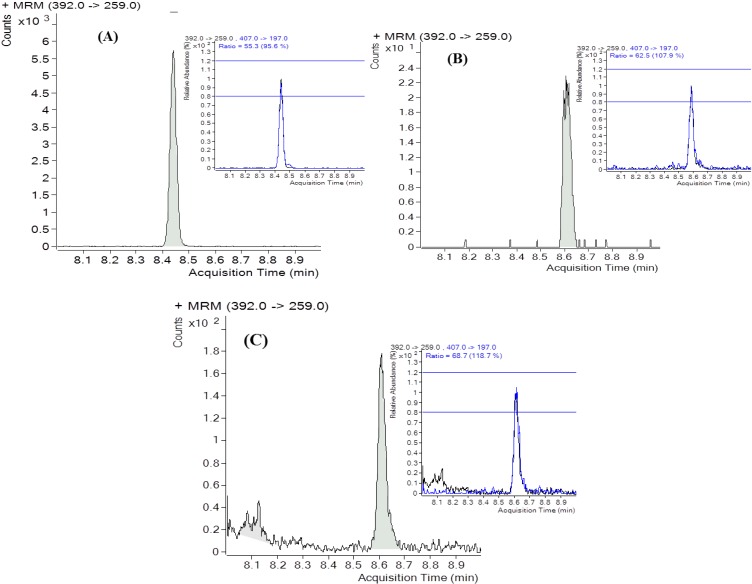
(**a**) SRM chromatograms of the naturally contaminated composite; (**b**) wheat-beer and (**c**) urine by deoxynivalenol at 122.6 µg/kg, 37.2 µg/kg and 20.8 µg/L, respectively.

Analyses in urine carried out by other authors have revealed the occurrence of DON in a high incidence of samples. For instance, Gratz *et al*. [30] reported an incidence of DON in all analyzed samples (*n* = 54). Similarly, DON incidences in urine samples of 33.3% (*n* = 27) and 67.6% (*n* = 34) were reported by Rubert *et al*. [31] and Turner *et al*. [21], respectively. In this work, a total of 1.87 L urine was collected as 24-hour urine volume and was in the normal excretion range according to sex and age [32]. DON was quantified at 17.5 ± 2.7 µg/g creatinine (*n* = 3) (equivalent to 18.8 ± 3.5 µg/L). No other mycotoxin was found in the urine sample being according with the results reported by Rubert *et al*. [31] and Warth *et al*. [33] who did not find neither any other trichothecenes nor zearalenone in the analyzed urine samples. GC-MS/MS chromatograms of naturally DON contaminated composite, beer and urine are shown in Figure 2. Urinary free DON levels of 18.8 ± 3.5 µg/L (equivalent to 35.2 ± 4.3 µg DON) was calculated in this study. Data found in literature were very similar. For instance, DON average contents of 20.4 µg /L were reported in an Austrian survey [27] (*n* = 27; incidence of 22%) and a range from 0.5 to 28.8 µg/L were reported in a French study (*n* = 76; incidence of 98.7%) [26].

### 2.5. Exposure Estimates

On the basis of the calculated data, DON daily intake was compared with the established PMTDI of 1 µg/kg bw [13]. The dietary exposure level to DON estimated in the present study was 0.683 µg/kg bw/day, lower than the value set by JECFA. Comparable values as regards dietary exposure to DON were reported in the second French total diet study [34] (mean: 0.379 µg/kg bw/day) as well as in a duplicate diet study carried out in Dutch young children (mean: 0.66 µg/kg bw/day) [35]. These results are also within the latest DON probable daily intake data (0.34 µg/kg bw/day) reported in the SCOOP task 3.2.10 derived from food analysis in Europe [36] and that reported by FAO/WHO (1.4 µg/kg bw/day) [37].

## 3. Experimental Section

### 3.1. Materials

Mycotoxin standards and metabolites namely de-epoxydeoxynivalenol (DOM-1), DON, 3-acetyldeoxynivalenol (3-ADON), fusarenon-X (FUS-X), diacetoxyscirpenol (DAS), nivalenol (NIV), neosolaniol (NEO), HT-2, T-2, zearalanone (ZAN), α-zearalanol (α-ZAL), β-zearalanol (β-ZAL), zearelenone (ZON), α-zearalenol (α-ZOL), β-zearalenol (β-ZOL) were obtained from Sigma-Aldrich (St. Louis, MO, USA). The derivatization reagent composed of BSA (*N*,*O*-bis(trimethylsilyl)acetamide) + TMCS (trimethylchlorosilane) + TMSI (*N*-trimethylsilyimidazole) (3:2:3) was purchased from Supelco (Bellefonte, PA, USA). Sodium dihydrogen phosphate and disodium hydrogen phosphate, used to prepare phosphate buffer, were acquired from Panreac Quimica S.L.U. (Barcelona, Spain).

Certified reference material BRM 003004 (artificially contaminated wheat, DON 1062 ± 110 µg/kg) was purchased from Biopure Referenzsubstanzen GmBH (Tulln, Austria).

All solvents, acetonitrile, hexane and methanol (HPLC grade), were purchased from Merck KGaA (Darmstadt, Germany). Anhydrous magnesium sulfate (thin powder) was obtained from Alfa Aesar GmbH & Co (Karlsruhe, Germany); sodium chloride was purchased from Merck and C18-E (50 μm, 65 A) was purchased from Phenomenex (Torrance, CA, USA). Picric acid (moistened with water, ≥98%) and creatinine standard were purchased from Sigma-Aldrich (St. Louis, MO, USA) whereas sodium hydroxide was acquired from BDH Prolabo—VWR International (Barcelona, Spain).

### 3.2. Standard Preparation

Individual stock solutions of all analytes were prepared at the same concentration (1000 mg/L) in methanol. The stock solutions were diluted with acetonitrile in order to obtain the appropriate multi-compounds working standard solutions (50 mg/L). All standards were stored in darkness and kept at −20 °C until the GC-MS/MS analysis. Calibration function of both neat solvent standards and spiked samples were established by plotting peak areas *versus* analyte concentrations in the measured solutions and performing linear regression. Linear range was tested from 0.1 to 100 µg/kg by spiking at eight concentration levels. In order to reveal the presence of matrix effect, matrix-matched calibration prepared by spiking extracts of blank samples with mycotoxins at similar concentrations than the calibration built in neat solvent without any matrix were compared. The slopes of the resulting linear calibration functions were compared and the signal suppression/enhancement due to matrix effects (ME) was determined as follows: $ME\;\left(\%\right)=\;\frac{Slope_{matrix-matched\;calibration}}{Slope_{standard\;in\;solvent}}\;x\;100$

### 3.3. Study Design and Sampling

In this 24 h preliminary study, a farinaceous-based diet was designed and conducted by a 26 year old, healthy male volunteer. Duplicate meals as prepared, served and consumed, based on a “duplicate plate” method were provided for subsequent individual analysis in sterile plastic food containers and kept with ice packs in a cooler until they were returned to the laboratory. The selection of food items analyzed in the present study was based on two criteria: first, the food must be identified by GEMS/Food as potential sources of mycotoxins and second, their level of consumption must exceed 1g/person day. Four complete meals were consumed during the day of the study as well as two beers in the afternoon. The food groups items selected were bread, wholegrain cereals, pasta, and wheat or barley-based beer. Food items such as pasta (spaghetti) and whole-wheat pasta (little stars) were cooked for 7 min in boiling water. A pasta/water ratio of 1:5 (*w*/*v*) was respected as recommended the preparation mode established in the food packaging labels. Salt and spices were also added as in regular cooking practices.

Upon arrival at the laboratory all meals were weighed and dried in an oven at 100 ± 4 °C to reach constant weight for subsequent mycotoxin analysis. The dried products obtained were thoroughly grounded and homogenized using a laboratory mill and kept at 4 °C under dark and dry conditions into a specific plastic food containers. Note that once dried and milled, all dried products were wheat flour-related foods. For the cooked pasta-based products, both dry cooked form and the dry uncooked ones were subjected to mycotoxin analysis to evaluate the percentage of mycotoxin reduction during the cooking.

A urine sample was collected as 24 h urine throughout the day of the study, and due to this, it was demonstrated that the main part of absorbed *Fusarium* toxins showed a rapid elimination within 24 h after ingestion [38]. Urine collected was stored at −20 °C until analysis. The 24 h period lasted from 8 am to 8 am on the next day to include the first morning urine. A written and approved informed consent was obtained from the volunteer. This project was approved by the University of Valencia Institutional human research Committee and the study purposes and procedures were justified and accepted for this study.

### 3.4. Composite Diet Sample

The composite diet was intended to be representative of 24 h duplicate diet collected and included all food items consumed over the monitoring period, without beverages. The dried food items were briefly homogenized, carefully mixed, and finally combined keeping the diet proportions.

### 3.5. Sample Preparation

Composite and individual meals were analyzed as described in detail elsewhere [39] in order to know the contribution of each food item. In brief, 5 g of homogenized sample was added to 25 mL distilled water and 7.5 mL of acetonitrile followed by the addition of 4 g of MgSO_4_ and 1 g of NaCl prior to be shaken vigorously and centrifuged for 3 min at 4000 rpm. Then the upper layer was submitted to a dispersive solid phase extraction (d-SPE) with a mixture of 900 mg of MgSO_4_ and 300 mg of C18 and centrifuged for 1 min at 1500 rpm. After centrifugation the liquid extract was separated from the solid salts and finally the extract was evaporated to dryness under nitrogen flow.

Beer samples were first completely degased by sonication for 15 min prior the analysis. A 10 mL portion was then used for the analysis. 5 mL of acetonitrile were added to the sample followed by the addition of the mixture of salts (MgSO_4_ and NaCl) and then submitted to a d-SPE as previously described.

The urine sample was first centrifuged at 4000 rpm for 5 min. A 10 mL portion of the centrifuged urine was then used for the subsequent analysis as indicated above.

All dry extracts were added with 50 µL of BSA + TMCS + TMSI (3:2:3) and the samples were left for 30 min at room temperature. The derivatized samples were diluted to 200 µL with hexane and mixed thoroughly on a vortex for 30 s. Then the diluted derivatized samples were added with 1 mL of phosphate buffer (60 mM, pH 7) shaken and the upper layers (hexane phases) were transferred to autosampler vials for the chromatographic analysis.

For quality control, certified reference material BRM 003004 (artificially contaminated wheat, DON 1062 ± 110 µg/kg) was used and included. Certified reference material was used as provided without further grinding. It was stored under the same conditions, extracted and determined with the same protocol as the analyzed samples. Each sample was analyzed in triplicate and measured in separate batches.

### 3.6. GC-MS/MS Method

A GC system Agilent 7890A coupled with an Agilent 7000A triple quadrupole mass spectrometer with inert electron-impact ion source and an Agilent 7693 autosampler (Agilent Technologies, Palo Alto, CA, USA) were used for MS/MS analysis. Chromatographic separation was achieved on a HP-5MS 30 m × 0.25 mm × 0.25 µm capillary column. One microliter of the final clean extract of mycotoxins was injected in splitless mode (equivalent to 25 mg of dried food matrix) at 250 °C in programmable temperature vaporization (PTV) inlet employing helium as carrier gas at fixed pressure of 20.3 psi. The oven temperature program was initially 80 ºC, and the temperature was increased to 245 °C at 60 °C/min. After a 3 min hold time, the temperature was increased to 260 °C at 3 °C/min and finally to 270 °C at 10 °C/min and then held for 10 min. Chromatographic analysis time was performed in 17 min, which reached the requirement for a high throughout determination [39].

The mass spectrometer operated in electron impact ionization (EI, 70 eV). The source and transfer line temperatures were 230 and 280 °C, respectively. The collision gas for MS/MS experiments was nitrogen, and the helium was used as quenching gas, both at 99.999% purity supplied by Carburos Metálicos S.L. (Barcelona, Spain). Data was acquired and processed using the Agilent Masshunter version B.04.00 software [39].

### 3.7. Calculation of DON Daily Intake

A deterministic approach was applied for the calculation of DON dietary exposure. The volunteer filled a 1-day food consumption record and was asked to provide his body weight (bw: 72 kg). DON daily intake, expressed as µg DON/kg bw, was calculated by combining food consumption (g foodstuff/kg bw/day) with DON food contamination (µg DON/g foodstuff) data. The contribution of each food to the average dietary exposure was also calculated.

### 3.8. Creatinine Analysis

Creatinine urinary levels were determined based on a spectrophotometric method slightly modified [40]. In summary, 3.5 mM picric acid was reacted with 1000 mM NaOH to form alkaline picrate. This solution was stored in the dark in an amber glass recipient. Alkaline picrate (1 mL) was reacted with 1 mL of diluted urine (1/10, *v*/*v*, in ultrapure water). The optical density was measured at 500 nm after 30 min using a Shimadzu mini 1240 spectrophotometer. Mycotoxin urinary concentrations were correlated to the creatinine content of a sample expressed as µg/g creatinine.

## 4. Conclusions

A total DON daily intake derived from the 24 h duplicate diet study amounted to 49.2 ± 5.6 µg whereas 35.2 ± 4.3 µg of DON were quantified in the urine collected in the same. DON incidence in urine of the participant confirms its exposure to DON and evidences the usefulness of DON and its metabolites in urine as a biomarker of exposure to such contaminants. The values of DON PDI estimated herein with the urinary biomarker approach matched quite well the intake derived from food analysis. DON data was further correlated to the established DON PMTDI value in order to obtain a risk characterization approach. DON daily intake represented a 68.3% of the established PMTDI. The obtained data from this preliminary study is subjected to intra- and inter-day variations. Therefore, this experiment needs to be extended to a larger group of individuals to investigate these variations and to elucidate the relation between ingested mycotoxins and excreted ones and their corresponding metabolites in humans. In this sense, the *in vitro* digestibility/metabolic models are very useful to complete the full-scale metabolism studies.
